# A role of the human thalamus in predicting the perceptual consequences of eye movements

**DOI:** 10.3389/fnsys.2013.00010

**Published:** 2013-04-23

**Authors:** Florian Ostendorf, Daniela Liebermann, Christoph J. Ploner

**Affiliations:** ^1^Department of Neurology, Charité - Universitätsmedizin BerlinBerlin, Germany; ^2^Berlin School of Mind and Brain, Humboldt Universität zu BerlinBerlin, Germany

**Keywords:** efference copy, corollary discharge, visual stability, prediction, thalamus, human, lesion, sensorimotor

## Abstract

Internal monitoring of oculomotor commands may help to anticipate and keep track of changes in perceptual input imposed by our eye movements. Neurophysiological studies in non-human primates identified corollary discharge (CD) signals of oculomotor commands that are conveyed via thalamus to frontal cortices. We tested whether disruption of these monitoring pathways on the thalamic level impairs the perceptual matching of visual input before and after an eye movement in human subjects. Fourteen patients with focal thalamic stroke and 20 healthy control subjects performed a task requiring a perceptual judgment across eye movements. Subjects reported the apparent displacement of a target cue that jumped unpredictably in sync with a saccadic eye movement. In a critical condition of this task, six patients exhibited clearly asymmetric perceptual performance for rightward vs. leftward saccade direction. Furthermore, perceptual judgments in seven patients systematically depended on oculomotor targeting errors, with self-generated targeting errors erroneously attributed to external stimulus jumps. Voxel-based lesion-symptom mapping identified an area in right central thalamus as critical for the perceptual matching of visual space across eye movements. Our findings suggest that trans-thalamic CD transmission decisively contributes to a correct prediction of the perceptual consequences of oculomotor actions.

## Introduction

Active perceptual exploration helps animals and humans to sample relevant aspects of the external world, but constantly changes sensory input. These self-generated changes in sensory input (so-called reafference) would severely impair coherent percepts when not properly distinguished from environmental changes. Forward models have been proposed as a candidate mechanism to anticipate the perceptual consequences of actions by an internal monitoring of corresponding motor commands (Wolpert and Miall, 1996). The existence of such internal monitoring signals had been proposed for a long time as an efficient means to disambiguate self-induced displacements of perceptual input from external changes in the outside world (Purkyně, 1825; Von Helmholtz, 1866). Important experimental support for internal monitoring processes was obtained by Von Holst and Mittelstaedt (1950) and Sperry (1950) who coined the hypothetical underlying signal “efference copy” or “corollary discharge” (CD), respectively.

Recently, single-unit recordings in non-human primates identified a CD pathway that conveys oculomotor monitoring information from brainstem structures to the frontal eye field (FEF) via central portions of the thalamus (Sommer and Wurtz, 2002). Additional experimental evidence suggests that information carried through this pathway bears direct functional relevance for visuomotor behavior: transient inactivation of this pathway on the thalamic level impaired oculomotor behavior in a task that required internal monitoring of saccade metrics (Sommer and Wurtz, 2002). Similar findings have been observed in patients with focal thalamic stroke (Gaymard et al., 1994; Bellebaum et al., 2005), suggesting that trans-thalamic CD is critical for accurate generation of rapid oculomotor sequences. These findings do however not directly address the question whether trans-thalamic CD signals are also involved in anticipating the perceptual changes imposed by saccades and whether CD signals may thus ultimately aid perceptual stability across eye movements.

Recently, we aimed to address this question in a single patient with a focal ischemic lesion of the right central thalamus (Ostendorf et al., 2010). The behavioral assessment of CD function in this patient seemed warranted because of the close anatomical overlap of his focal lesion with the homologous thalamic site in the monkey brain at which CD signals had been recorded (Sommer and Wurtz, 2002, 2004). We used a simple visuomotor task to assess a possible deficit in the perceptual matching of space across eye movements: subjects were instructed to report the apparent direction of an unpredictable target displacement that happened in temporal contingency with a saccadic eye movement to this target stimulus. Attenuation of motion perception during saccades (Burr et al., 1982) limits the usefulness of intrasaccadic motion cues to guide this perceptual decision. Hence, surprisingly large object displacements can escape conscious detection when they take place during saccadic eye movements, a phenomenon called saccadic suppression of displacement (SSD; Bridgeman et al., 1975). However, small modifications of the original task can lead to dramatic performance improvements in healthy subjects (Deubel and Schneider, 1994; Deubel et al., 1996): a short blanking of the target reverses SSD to high perceptual sensitivity for displacement detection that can even exceed performance under steady fixation (Deubel et al., 1996). Thus, a faithful representation of target position is apparently retained across eye movements and can, at least under certain conditions, be combined with accurate and precise oculomotor monitoring information to effectively guide perceptual judgments.

Compared to age-matched control subjects, we observed a lateralized deficit for this task variant in the patient, manifesting as inaccurate matching of locations across eye movements (Ostendorf et al., 2010). He showed a systematic bias of perceptual reports toward apparent backward displacements that was consistent with an internal underestimation of eye movement amplitudes. Side and sign of this perceptual deficit were identical to additional impairments observed for the generation of rapid saccade sequences, pointing toward a common disruption of internal monitoring underlying both behavioral deficits. Moreover, the putative deficit in eye movement monitoring led to a systematic dependency of perceptual decisions on saccadic errors in the patient (Ostendorf et al., 2010). While normal subjects can reliably predict trial-to-trial variations in eye movement targeting and anticipate the associated perceptual mismatches (Collins et al., 2009), he systematically misattributed self-induced visual errors to external stimulus changes (Ostendorf et al., 2010). Taken together, behavioral deficits in this patient were consistent with an incomplete and noisy CD signal, leading to uncertain and hypometric estimates of executed eye movements.

Here, we aim to address specificity and generalizability (Robertson et al., 1993) of our previous findings by probing perceptual performance in a larger sample of 14 patients who sustained focal thalamic lesions from ischemic stroke in different portions of the thalamus. As in our case study (Ostendorf et al., 2010), we used the original intrasaccadic displacement task in which SSD is expected to appear (Bridgeman et al., 1975) and the task variant proposed by Deubel et al. (1996) in which high perceptual sensitivity in normal subjects has been demonstrated repeatedly (Deubel et al., 1996; Collins et al., 2009). We compared perceptual performance in the patient group with a sample of control subjects in these two task variants. We capitalized on intra-individual differences between task conditions and saccade directions (Bellebaum et al., 2005) to identify deficits in the trans-saccadic matching of visual space in individual patients. Beyond standard groupwise comparisons, the acquisition of high-resolution imaging data at the time of behavioral testing allowed us to perform voxel-based lesion-symptom mapping (Rorden and Karnath, 2004) in order to identify thalamic regions critical for task performance.

## Methods

### Subjects

Fourteen patients with focal lesions of the thalamus [mean age ± standard deviation (SD), 40.6 ± 9.1 years; five females] participated in this study. Patients were recruited from the Department of Neurology, Charité - Universitätsmedizin Berlin, Germany and were part of a patient cohort that had participated in a recent neuropsychological study (Liebermann et al., 2013). Twenty healthy subjects (38.8 ± 7.8 years; eight females) served as controls. Handedness was assessed by Edinburgh-Handedness-Inventory (Oldfield, 1971) with a laterality quotient of ≥40 and ≤40 denoting right and left-handedness, respectively. In the patient group, 13 subjects were right-handed, one left-handed and none ambidextrous (mean laterality quotient ± SD, 64.2 ± 36.2). 17 control subjects were right-handed, two left-handed, and one ambidextrous (mean laterality quotient ± SD, 68.3 ± 55.1). Average years of education (±SD) were 14.3 (±2.8) in patients and 16.1 (±2.3) in control subjects. No significant differences emerged for these demographic measures between patients and the control subjects. Control subjects had no history of neurological or psychiatric disorders and all but one were naive with respect to the purpose of the study. Informed consent was obtained from all subjects before participation in the study, which was approved by the local Ethics Committee (Charité - Universitätsmedizin Berlin, Campus Mitte, Germany). Apart from a slight right-sided hemiparesis accompanied by prickling paresthesia in one patient (P11), neurological examination was normal in all patients at the time of testing (see Table 1 for initial symptoms of individual patients).

**Table 1:** **Demographic and lesion characteristics of patients**.

**Patient**	**Age**	**Sex**	**Years of education**	**IQ**	**TSL (months)**	**Lesion side**	**Lesion vol. (cm^3^)**	**Initial symptoms**
1	51	F	9	94	22	R	0.13	Left hemiparesis and hypesthesia
2	45	M	13	130	0.25	L	0.06	Right hyp-/paresthesia
3	40	M	18	n/a	12	L	0.07	Right hemiparesis, diplopia
4	45	M	18	118	1	L	0.03	Anomic aphasia, dizziness
5	36	M	13	124	29	B (R>L)	0.03	Diplopia, anomic aphasia
6	43	F	13	n/a	12	B (L>R)	0.36	Vigilance disturbance, aphasia
7	31	M	13	112	8	R	0.12	Left hemiparesis and paresthesia, headache
8	53	M	12	100	1	R	2.35	Left hemiparesis, ataxia, vigilance disturbance, dizziness
9	25	F	13	101	0.5	R	0.21	Headache, nausea
10	37	M	13	118	10	R	0.2	Left hemiparesis, amnesia
11	57	F	13	118	60	L	0.15	Right hemiparesis and paresthesia^*^
12	39	M	18	130	11	R	0.26	Diplopia, dysarthria, vertigo
13	32	F	18	124	2	L	0.2	Right hemiparesis, diplopia, vertigo
14	34	M	16	118	45	B (R>L)	1.13	Right hemiparesis, diplopia, dysarthria, loss of consciousness

Asterisk indicates persistent symptom (only patient P11).

Abbreviations: Lesion vol., lesion volume; TSL, time since lesion.

### Imaging and lesion reconstruction

Imaging and lesion reconstruction was identical to Liebermann et al. (2013). In brief, structural imaging was performed on a clinical whole-body scanner (Magnetom Vision, Siemens) at 1.5 T. For reconstruction of lesions, a three-dimensional dataset was acquired, using a magnetization prepared rapid acquisition gradient-echo imaging sequence (MPRAGE, isotropic resolution 1 mm). To screen for additional extra- and intrathalamic lesions at the time of testing, axial images of the whole brain and coronal images of the thalamic region were acquired using a T2-weighted turbo inversion recovery magnitude sequence (whole brain and thalamus, voxel-size 0.91 × 0.9 × 5 mm and 0.95 × 0.9 × 2 mm, respectively). High-resolution imaging revealed no further lesions except for single lacunar lesions in four patients [left cerebellum, lobule VI (patient P6) and lobule VIIIb (patient P3), right cerebellum, lobule VI (patient P5), and genu of corpus callosum (patient P8)]. In addition, the thalamic lesion of one patient extended slightly into the right hypothalamus (patient P13).

**Figure 1:**
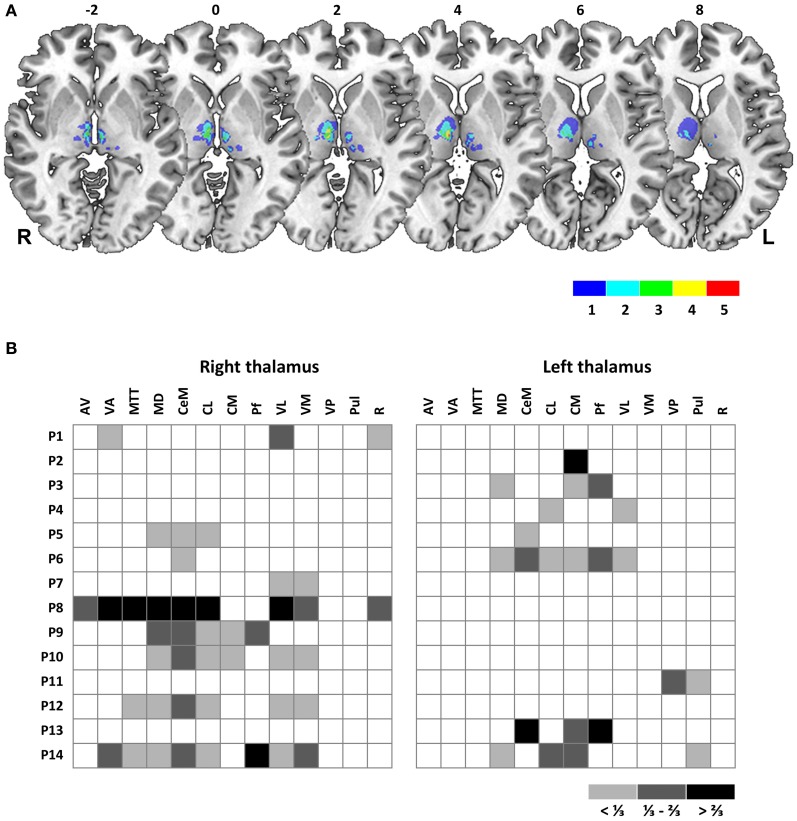
**(A)** Overlay plot of thalamic lesions in patient sample. Lesion volumes of all 14 patients are plotted on axial sections of a MNI brain template with numbers denoting z-coordinates in MNI space. Different colors denote the number of overlapping lesions per voxel, ranging from 1 to a maximum of 5 individual lesion volumes. Image display follows radiological convention with right hemisphere (R) shown on left side of picture. L, Left; R, Right hemisphere. **(B)** Affected thalamic nuclei, as determined by co-registration of individual MRI to an established atlas of the human thalamus (Morel, 2007). Relative lesion extent for a given nucleus is displayed in three increments: White, not affected, light gray, less than 1/3 of total volume affected, dark gray, between 1/3 and 2/3 affected, black, more than 2/3 affected. Patients are labeled by ascending numbers. Abbreviations: AV, anteroventral nucleus; VA, ventral anterior nucleus; Mtt, mamillothalamic tract; MD, mediodorsal nucleus; CeM, central medial nucleus; CL, central lateral nucleus; CM, centromedian nucleus; Pf, parafascicular nucleus; VL, ventral lateral nucleus; VM, ventral medial nucleus; VP, ventral posterior nucleus; Pul, pulvinar; R, reticular nucleus.

Individual brain scans were spatially normalized using MATLAB (The MathWorks, Natick, MA, USA) and the Statistical Parametric Mapping package (SPM5, Wellcome Department of Imaging Neuroscience, London, http://www.fil.ion.ucl.ac.uk/spm). Individual MRI data sets were normalized to a T1 Montreal Neurological Institute (MNI) template provided with SPM by using the unified segmentation and normalization function. This method has recently been demonstrated to provide reliable normalization of focally lesioned brains to template images (Crinion et al., 2007), although cost function masking might still be recommended for larger lesions (Andersen et al., 2010). For identification of affected thalamic nuclei in individual patients, lesions were co-registered to an atlas of the human thalamus (Morel, 2007). Coronal reconstructions from MRI data sets were evaluated against corresponding atlas plates. Relative lesion extent for a given thalamic nucleus was rated in three increments (less than 1/3, between 1/3 and 2/3, more than 2/3 of nucleus volume affected, see Figure 1B). Lesion overlap and subtraction plots (Rorden and Karnath, 2004) were generated on a group level for further lesion-to-symptom mapping. For this analysis, lesions were manually traced in normalized three-dimensional space with MRIcron software (version as of December 2012, www.mccauslandcenter.sc.edu/mricro/mricron/index.html). Estimation of lesion volume and lesion overlap and subtraction analyses (Rorden and Karnath, 2004) were conducted with resulting volumes of interest (VOIs) in MRIcron. For further statistical analysis we used non-parametric voxel-based lesion-symptom mapping as implemented in NPM, which is part of the MRIcron software package. Only voxels that were lesioned in at least 2 patients were included in this analysis.

### Experimental set-up and task

The intrasaccadic displacement task was identical to Ostendorf et al. (2010). Stimuli were presented on a 22-in. CRT-monitor (screen resolution, 1024 × 768 pixels; refresh rate, 110 Hz) at a viewing distance of 50 cm. Subjects' heads were stabilized by a head- and chinrest. Eye movements were recorded with high-speed video-oculography (Sensomotoric Instruments; sampling rate, 500 Hz) of the right eye. Experiments were carried out in an otherwise darkened room. Subjects completed the experiments in multiple test sessions on different days. All stimuli were white (luminance, 56.5 Cd/m^2^) and presented on a homogenous gray background (luminance, 13.1 Cd/m^2^).

**Figure 2:**
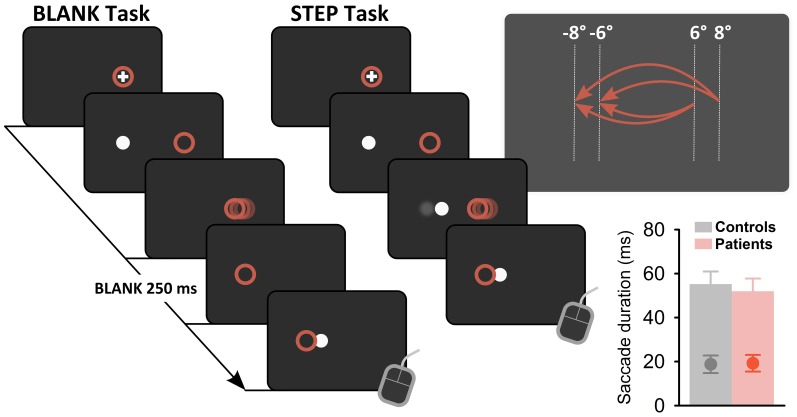
**Task schematic.** In both task conditions, subjects fixated on a fixation cross, presented on right or left side of screen. After a variable foreperiod (1600–2400 ms), the fixation cross was extinguished and a target stimulus (white dot) presented on the other side of screen. Subjects performed a saccadic eye movement to this target (actual eye position, red circles). Saccade onset triggered a stimulus change: in the BLANK condition, the target was switched off and reappeared 250 ms later at a displaced position. In the STEP condition, saccade onset triggered a direct displacement of the target. In both conditions, subjects were instructed to indicate the apparent displacement direction by means of a button press at trial end. Upper panel on right side shows possible start and end positions of requested saccades for a block of leftward saccade direction, yielding stimulus amplitudes of −12, −14, and −16°, respectively. Lower panel on right side shows average saccade duration for control subjects (gray bar) and patients (red bar) with average trigger times for gaze contingent stimulus changes superimposed (gray and red circles, respectively). Error bars denote ±1 SD.

Trials started with presentation of a fixation cross (extent, 0.5°) at 6 or 8° left or right from screen center (see Figure 2 for task schematic). After a variable foreperiod (1600–2400 ms), the fixation cross was switched off and a target cue (diameter, 0.5°) was presented at the other screen side at 6 or 8° eccentricity, respectively. Subjects were instructed to perform a saccadic eye movement toward this target, which was switched off during saccade execution and reappeared either directly (STEP condition) or after a temporal gap of 250 ms (BLANK condition) at an unpredictable position. Target displacement for a given trial was adapted by three independent, randomly interleaved staircases with a constant step size of 3° in the STEP task (BLANK task, 1.5°). Specifically, when the subject indicated a target displacement to the left for a given displacement level, the next displacement level for a given staircase would be shifted by 3° (BLANK task, 1.5°) to the right, i.e., staircases followed a one-up, one-down logic. Staircases started at a displacement level of 7° (GAP task, 3.5°) right- and leftward and 0° (no displacement) with respect to initial target position. Interleaved displacement levels for the three staircases enabled sampling the point of subjective target constancy with a resolution of 1° (BLANK task, 0.5°) while collecting a sufficient number of trials at higher confidence levels. In both conditions, subjects reported the apparent jump direction by pressing one of two manual response keys. Response registration was limited to maximally 5 s and the target was switched off when a key press was recorded or maximum response time had elapsed. The screen was then blanked for 1600 ms and a next trial started. Saccade direction was fixed within five to six blocks of 24 trials each.

### Data analysis

Eye movement data were low-pass filtered, visualized, and analyzed in Matlab by using the ILAB toolbox (Gitelman, 2002) and self-written routines. Saccade onset and offset were determined by a fixed velocity criterion (threshold, 30°/s). Start and end positions were determined as fixation periods preceding saccade onset and following saccade end. We ensured that intrasaccadic target displacements occurred during the first half of the saccadic eye movement [mean delay after saccade onset (±SD), 19 (±4) ms; see lower right panel in Figure 2]. Cumulative gaussians were fitted to the perceptual response data in Matlab by using psignifit, a toolbox that implements the maximum-likelihood method described by Wichmann and Hill (2001). From psychometric functions, we determined the point of subjective target stationarity (PSS) as a measure of bias in perceptual reports and the standard deviation of the fitted cumulative gaussian as a measure of just-noticeable difference (JND). For easier comparison of perceptual performance between conditions and subjects, we converted the psychometric function to percent correct (discarding trials with null displacement) and averaged resulting values for corresponding negative and positive displacement levels. We determined a perceptual threshold as the absolute displacement needed to achieve correct responses in 75% of trials (Ostendorf et al., 2010, 2012). Statistical analyses of oculomotor and perceptual response data were performed in SPSS, version 19.0 (IBM, Armonk, NY, USA). Tests on group differences between single measures of interest were performed by using *t*-tests and Mann–Whitney *U*-tests, respectively. Group differences were evaluated by repeated measures ANOVA with factors GROUP (control vs. patient group), CONDITION (BLANK vs. STEP), and SIDE (rightward vs. leftward saccades). Significance threshold was set at *P* = 0.05.

## Results

### Lesion characteristics

In our sample, 6 patients (43%) sustained a unilateral right, 5 patients (36%) a unilateral left and 3 patients (21%) a bilateral thalamic lesion. Time since lesion varied from 25–60 months (mean, 15.3 months), lesion volume ranged from 0.03–2.35 cm^3^ (mean, 0.38 cm^3^). Thalamic lesions predominantly involved the ventral, medial, and lateral parts of the thalamus (see Figure 1A for an overlap of individual lesion volumes). Most commonly affected nuclei were mediodorsal (MD, 9 patients, 64.3%) and ventral lateral (VL, 8 patients, 57.1%) as well as intralaminar nuclei: the central medial (CeM) and central lateral (CL) nuclei and the centromedian-parafascicular complex (CM-Pf) were affected in 8, 8, and 7 patients (57.1, 57.1, and 50%), respectively. By contrast, the anterior and posterior nuclei were largely spared. A display of affected nuclei for individual patients is given in Figure 1B. Further patients' characteristics are summarized in Table 1 (with premorbid intelligence level estimated by the MWT-B, a German equivalent to the National Adult Reading Test).

### Oculomotor performance

Patients and control subjects did not differ in terms of basic oculomotor performance. The grand average of saccadic reaction times (SRT) was 202 ms and repeated-measures ANOVA revealed no significant effect of SIDE [*F*_(1, 32)_ = 1.99, *P* = 0.17], CONDITION [*F*_(1, 32)_ = 1.18, *P* = 0.29], or GROUP [*F*_(1, 32)_ = 0.45, *P* = 0.51] and no significant interaction between these factors (all *P* ≥ 0.08). We assessed amplitude errors of first saccades as amplitude gain (saccade amplitude divided by stimulus amplitude). A repeated-measures ANOVA on saccade gain revealed a significant effect of SIDE [rightward vs. leftward saccade direction, *F*_(1, 32)_ = 43.0, *P* < 10^−6^] with on average slightly more hypometric leftward saccades [average gain for leftward (rightward) saccades, 0.90 (0.94)]. This finding is readily explained by monocular recording of the right eye in our study (Collewijn et al., 1988). No effect of GROUP [controls vs. patients, *F*_(1, 32)_ = 0.79, *P* = 0.38] or CONDITION [BLANK vs. STEP, *F*_(1, 32)_ = 1.57, *P* = 0.22] and no interactions between factors were observed (all *P* ≥ 0.15).

We also analyzed the systematic and variable error of saccade landing positions. Paralleling the analysis of amplitude gain, repeated-measures ANOVA revealed a significant effect of SIDE on systematic targeting errors [*F*_(1, 32)_ = 41.8, *P* < 10^−6^] with leftward saccades falling systematically shorter of target position [mean targeting error for leftward (rightward) saccades, −1.59° (−0.87°)]. A marginally significant effect of CONDITION was observed [*F*_(1, 32)_ = 3.9, *P* = 0.06], but no effect of GROUP [*F*_(1, 32)_ = 1.8, *P* = 0.19] and no interactions between these factors (all *P* ≥ 0.09) were noted. We assessed the variable error of saccade landing positions as standard deviation of targeting errors. For this measure, a repeated-measures ANOVA showed a significant effect of SIDE [*F*_(1, 32)_ = 9.7, *P* = 0.004] and GROUP [*F*_(1, 32)_ = 5.2, *P* = 0.03], with a slightly larger targeting scatter for leftward saccades [standard deviation of targeting error for leftward (rightward) saccades, 1.06° (0.93°)] and for the patient group [patients (controls), 1.11° (0.88°)], respectively. No effect of CONDITION [*F*_(1, 32)_ = 1.7, *P* = 0.21] and no significant interactions between these factors (all *P* ≥ 0.49) were observed.

### Perceptual performance

**Figure 3:**
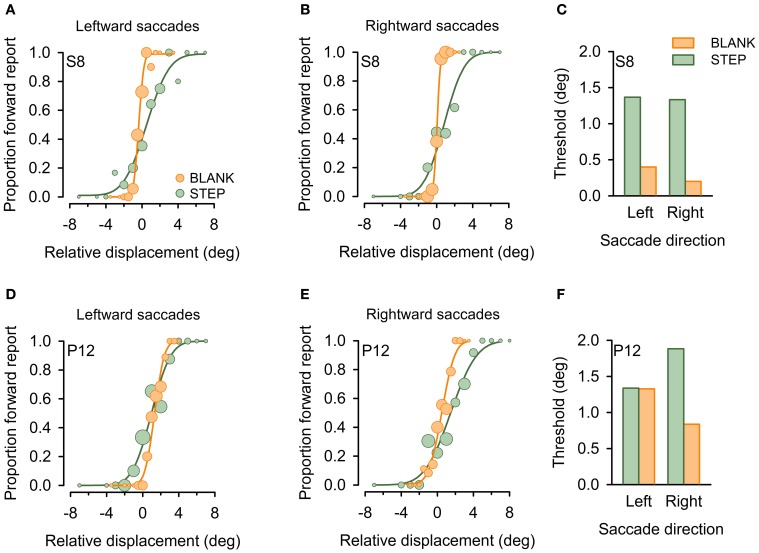
**Example psychometric functions in individual subjects.** Data are shown for one healthy control subject (S8, **A–C**) and a patient with a focal lesion in the right central thalamus (P12, **D–F**) for the STEP condition (green) and the BLANK condition (orange). Circles denote proportion of trials in which subjects reported an apparent stimulus jump in saccade direction (forward), plotted against relative displacement level. Negative values refer to target displacements against saccade direction. Circle size represent the number of trials for a given target jump. Cumulative gaussians were fitted to perceptual response data separately for leftward **(A,D)** and rightward **(B,E)** saccades. (**C** and **F**) display resulting detection thresholds, calculated as absolute displacement needed to achieve 75% correct responses.

Figure 3 plots exemplary results for one control subject (S8, Figures 3A,B) and one patient with a lesion in the right thalamus (P12, Figures 3D,E). Replicating previous findings (Deubel et al., 1996), psychometric functions in the control subject reveal improved performance for the BLANK compared to the STEP condition for both saccade directions with more accurate (i.e., less-biased PSS) and more precise performance (i.e., steeper slope of psychometric functions and corresponding lower JND). This is reflected in a strong improvement of detection threshold for the BLANK condition for both saccades direction [see Figure 3C; average threshold for BLANK (STEP), 0.3° (1.35°)]. The same pattern can be observed for rightward saccades in the patient, with threshold improving from 1.88° in the STEP condition to 0.84° in the BLANK condition. By contrast, no improvement is apparent for leftward saccades with almost identical thresholds in the STEP (1.34°) and BLANK (1.33°) task. Psychometric functions (Figure 3D) demonstrate that the lack of threshold improvement for leftward saccades was mainly caused by a larger forward bias of the psychometric function [PSS in the STEP (BLANK) condition, 0.98° (1.32°)].

**Figure 4:**
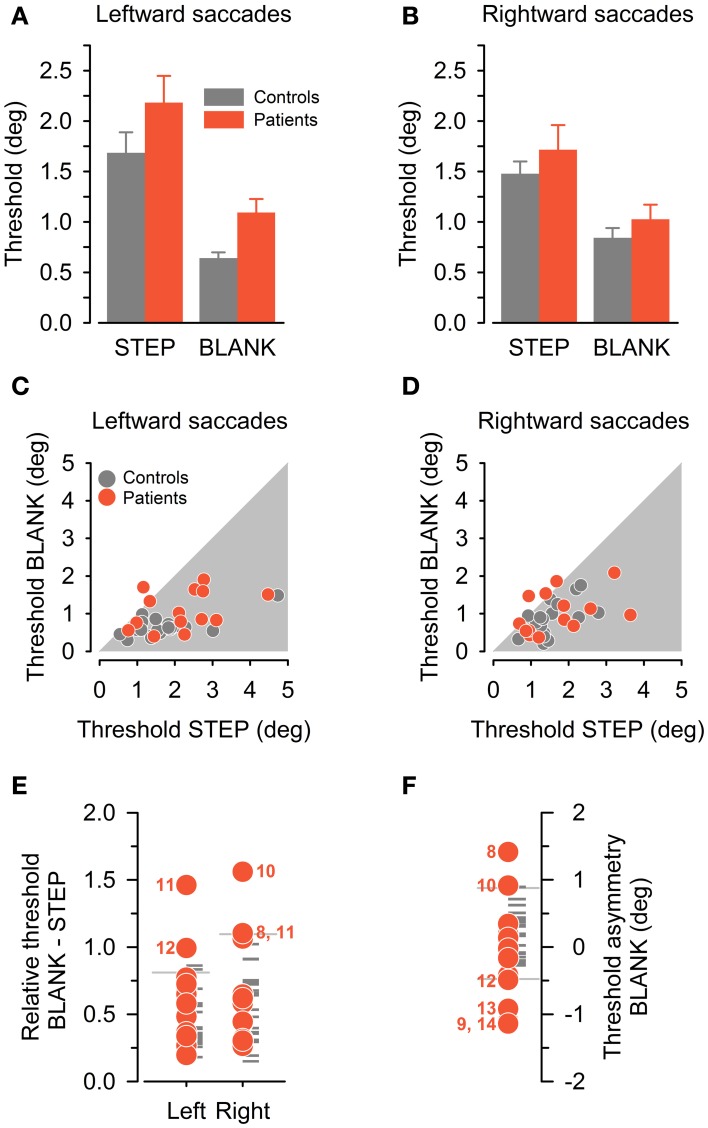
**Perceptual performance in control subjects (gray color) and patients (red color). (A,B)** Group average detection thresholds (mean ± SEM) are plotted separately for leftward **(A)** and rightward **(B)** saccade direction and for the STEP and BLANK condition. **(C,D)** Individual detection thresholds in BLANK task, plotted against corresponding threshold in STEP task, separately for leftward **(C)** and rightward **(D)** saccade direction. **(E)** Detection thresholds in BLANK task relative to STEP task, separately for leftward and rightward saccades. Reference lines denote corresponding control subjects' mean plus 1.96 SD. **(F)** Asymmetry of detection threshold in BLANK task for rightward minus leftward saccade direction. Reference lines denote the ±1.96 SD-interval of control subjects' sample. In **(E)** and **(F)**, patients with relative thresholds beyond 1.96 standard deviation of the controls' mean are marked with their ID number.

Figure 4 plots group average thresholds for the STEP and BLANK condition. Inspection of the graphs suggests a robust effect of task condition with improved perceptual performance in the BLANK compared to the STEP condition (average improvement, 37 and 49% for patients and control subjects, respectively). For both patient and control group, this improvement in the BLANK condition is on average larger for leftward than rightward saccades (average improvement 51 vs. 37%, respectively). Repeated-measures ANOVA confirmed a main effect of CONDITION [BLANK vs. STEP condition, *F*_(1, 32)_ = 65.3, *P* < 10^−8^], no effect of SIDE [rightward vs. leftward saccade direction, *F*_(1, 32)_ = 1.5, *P* = 0.24], but a significant interaction between CONDITION and SIDE [*F*_(1, 32)_ = 7.1, *P* = 0.01]. Furthermore, average thresholds in patients are higher compared to the control sample in all conditions [average thresholds in patients (controls), 1.5° (1.16°)]. A significant main effect of the between-subject factor GROUP [control subjects vs. patients, *F*_(1, 32)_ = 4.7, *P* = 0.04] was confirmed statistically with no interaction of GROUP with CONDITION, SIDE, or CONDITION × SIDE (all *P* ≥ 0.24).

Individual thresholds exhibited considerable variability in our comparatively large and heterogeneous sample of control subjects. This can be appreciated by inspection of Figures 4C,D, plotting individual thresholds in the BLANK condition against corresponding thresholds in the STEP condition. Most data-points are located below unity line (i.e., within gray-shaded area), confirming on an individual level the perceptual benefit of the BLANK manipulation. By contrast, one patient apparently exhibited a paradoxical deterioration of thresholds in the BLANK condition for leftward saccades, three patients for rightward saccades.

### Individual case analysis

For further analysis of individual perceptual performance in patients, we capitalized on individual threshold differences between the two task conditions (BLANK vs. STEP) and saccade directions in the BLANK task (rightward vs. leftward), similar to previous approaches in the literature (Bellebaum et al., 2005). In these analyses, we scored performance as deficient if a patient's threshold was found to be beyond ±1.96 SD of the control subjects' mean. Figure 4E plots perceptual thresholds in the BLANK condition relative to the corresponding threshold in the STEP condition (i.e., a relative threshold of unity indicates identical thresholds in BLANK compared to STEP task, a threshold below unity a proportional improvement). For all but one subject and saccade direction, relative thresholds of control subjects are indeed below unity, indicating a relative improvement in perceptual performance.

Compared to the control group, four of our patients exhibited impaired performance in this analysis, one for leftward, two for rightward saccade directions, and one for both saccade directions (abnormal thresholds are marked by corresponding patient ID number). This includes patient P10, previously reported as a case study (Ostendorf et al., 2010). For all but one of the five affected saccade directions, the missing BLANK effect was caused by a systematic forward shift of the psychometric function in the BLANK compared to the STEP condition (cf. psychometric function for leftward saccades in exemplary patient, Figure 3D). However, no obvious pattern emerged concerning lateralization of the behavioral deficit with respect to lesion side: three patients sustained right sided thalamic damage with behavioral deficits manifesting ipsilateral to lesion side in two patients (P8, 10) and contralateral to lesion side in one case (P12). One patient (P11) sustained a left-sided lesion and exhibited a bilateral behavioral deficit.

In a next analysis step, we compared individual performance in the BLANK task for rightward vs. leftward saccades. Figure 4F plots individual threshold asymmetry sores (calculated as a subtraction of leftward from rightward thresholds). Thresholds in the control group were found to be symmetrical on average, with slightly higher thresholds for rightward saccades [average threshold asymmetry in control group (±SD), 0.20° (±0.35°)]. Compared to the control group, six patients exhibited an abnormal threshold asymmetry, two with higher thresholds for rightward saccades and four with higher thresholds for leftward saccades (abnormal threshold asymmetries are marked by corresponding patient ID number).

Asymmetry of BLANK thresholds was primarily driven by systematic biases of the psychometric function: for three patients (P8, 10, 12), abnormal asymmetry was caused by a forward bias for one saccade direction, for the other three patients (P9, 13, 14), a backward bias for one saccade direction was the main factor driving the asymmetry. These differences in perceptual biases for rightward vs. leftward saccade direction could not simply be attributed to corresponding differences in targeting error of corresponding saccades (Pearson's correlation, *P* = 0.21). No consistent association of lesion side and the behavioral deficit emerged: four patients sustained right-sided thalamic damage with higher thresholds manifesting ipsilateral to lesion side in two patients (P8, 10) and contralateral to lesion side in the other two (P9, 12). One patient with a left-sided lesion exhibited increased thresholds ipsilateral to lesion side (P13) and one patient with a bilateral, predominantly right-sided lesion exhibited increased thresholds for leftward saccades as well (P14).

As a final behavioral probe of internal monitoring, we analyzed a possible dependency of perceptual report on saccade targeting error: with only unreliable CD information available, the correct attribution of visual errors after saccade execution to either self-induced targeting errors or external stimulus jumps should be more difficult. Perceptual reports should increasingly become contaminated by oculomotor noise. Previous studies showed that normal subjects can indeed take their own trial-by-trial oculomotor imprecision into account for the perceptual matching of stimulus locations across eye movements (Collins et al., 2009; Ostendorf et al., 2010). For analysis, we binned perceptual data in the BLANK condition on an individual basis according to the oculomotor targeting error. As in Ostendorf et al. (2010), we used eight bins of equal sample size, separately for rightward and leftward saccades.

**Figure 5:**
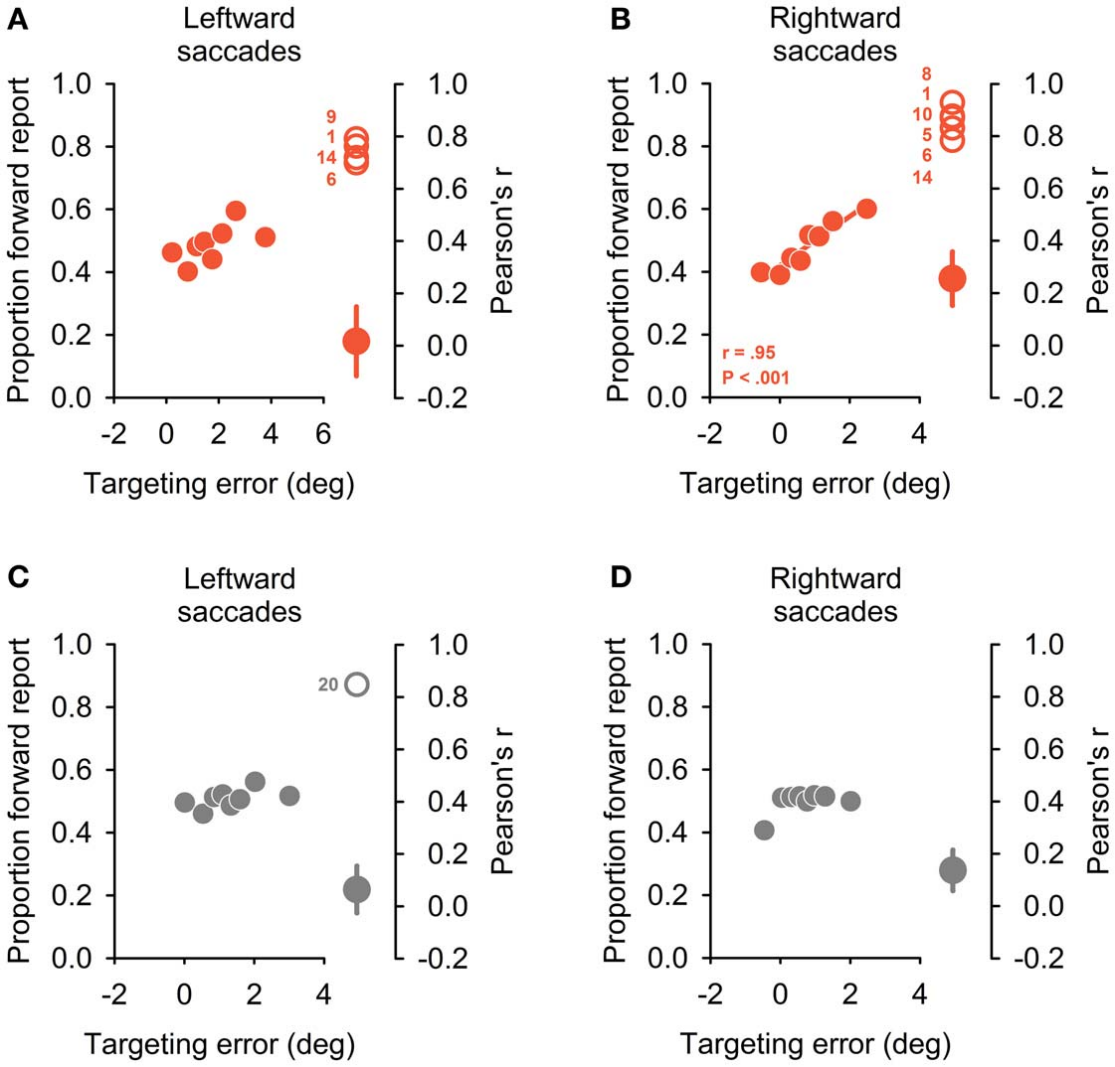
**Dependency of perceptual reports on oculomotor targeting errors.** On left side of the four panels, the proportion of forward reports is plotted against relative oculomotor error for eight error bins of equal sample size. Positive targeting errors refer to hypometric primary saccades. Plots depict the group average for the patient group **(A,B)** and control sample **(C,D)**, separately for leftward **(A,C)** and rightward **(B,D)** saccade directions. A significant correlation emerged only for rightward saccades in patients **(B)** and the inset indicates correlation coefficient and corresponding *P*-value (Pearson correlation). Right side of the four panels depicts significant individual correlation coefficients (open circles) together with average coefficient for the rest of the patient group **(A,B)** or control subject sample **(C,D)**, respectively (filled circles, mean ± SEM). Significant correlations emerged in four patients and one control subject for leftward and in 6 patients and no control subject for rightward saccades.

No significant correlation emerged between binned oculomotor targeting error and perceptual reports for all but one control subject and saccade direction (Pearson's correlation; see Figures 5C,D, plotting group averages of perceptual report bins against corresponding quantized targeting error). By contrast, significant correlations emerged for seven patients in this analysis (Figures 5A,B): one patient exhibited a significant correlation for leftward saccades, three patients for rightward saccades ad three patients for both saccade directions. This dependency of perceptual performance on targeting errors could not be explained by deficient oculomotor performance: no significant differences emerged when comparing systematic and variable targeting errors in affected vs. non-affected patients (for both BLANK and STEP condition and leftward and rightward saccades, all *P* ≥ 0.26). Again, as with the two other behavioral measures reported above, no obvious pattern of lateralization emerged with respect to thalamic lesion side: of the seven patients performing deficiently, two patients with right-sided lesions exhibited the behavioral deficit for ipsilateral saccade direction (P8, 10), one patient with a right-sided lesion showed it for contralateral saccade direction with respect to lesion side (P9). One patient with a bilateral, predominantly right-sided lesion, showed a significant correlation for rightward saccades (P5). Two of the three patients exhibiting a behavioral deficit for both saccade directions sustained bilateral lesions (P6, 14); one had a right-sided lesion (P1).

### Lesion-symptom mapping

The observed differential deficits in the perceptual matching of visual space across eye movements could not simply be explained by lesion acuity. For none of the three behavioral measures did time since lesion significantly differ between impaired und unimpaired patients (all *P* ≥ 0.4). Likewise, no significant association between the presence of extrathalamic lesions and behavioral deficits emerged for the three scores (Pearson's chi-square, χ^2^ ≤ 0.31, *P* ≥ 0.58).

**Figure 6:**
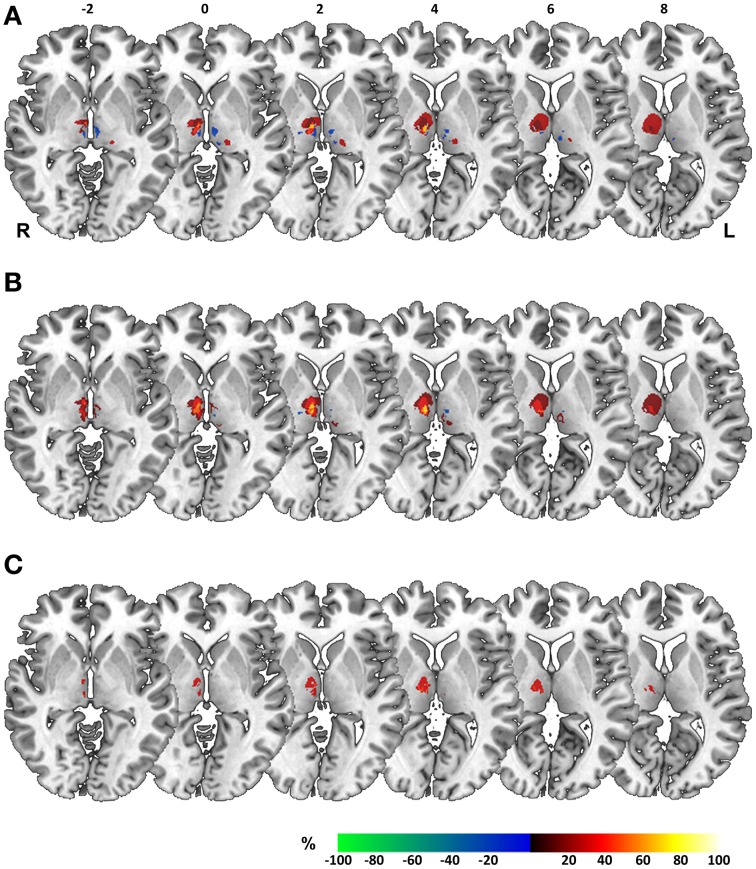
**Overlay lesion plots.** Overlay plots of superimposed lesions of impaired minus unimpaired patients, shown on axial sections of a MNI brain template with numbers denoting z-coordinates in MNI space. Colors denote the percentage of overlapping lesions of patients performing deficiently for a specific perceptual measure after subtraction of unimpaired patients. Overlap-subtraction plots are thresholded at 15%, i.e., only regions lesioned more or less frequently in at least 15% of the impaired vs. unimpaired patients are shown in this plot. Behavioral measures for construction of overlay plots comprised **(A)** abnormal thresholds in BLANK relative to STEP task, **(B)** threshold asymmetry in the BLANK task for right- vs. leftward saccades. and **(C)** a systematic dependency of perceptual reports on oculomotor targeting errors. L, Left; R, Right hemisphere.

Acquisition of high-resolution MR image datasets at the time of testing allowed for further investigation of a possible common lesion zone in patients performing deficiently in the perceptual matching of space across eye movement. To this end, we performed voxel-based lesion-symptom mapping in our patient sample, taking the three behavioral scores elaborated on above as dichotomous classifiers to rate patients as impaired versus unimpaired. Figure 6 shows overlay plots of superimposed lesions of patients classified as impaired for a specific score minus those classified as unimpaired. Such an overlap-subtraction logic aims to reveal regions that are critical for task performance while controlling for the effect of commonly affected, but innocent bystander regions (Rorden and Karnath, 2004). Overlay lesion plots are shown separately for (1) abnormal thresholds in BLANK relative to STEP task (Figure 6A), (2) abnormal threshold asymmetry in BLANK task (Figure 6B), and (3) a dependency of perceptual reports on oculomotor targeting errors (Figure 6C).

**Figure 7:**
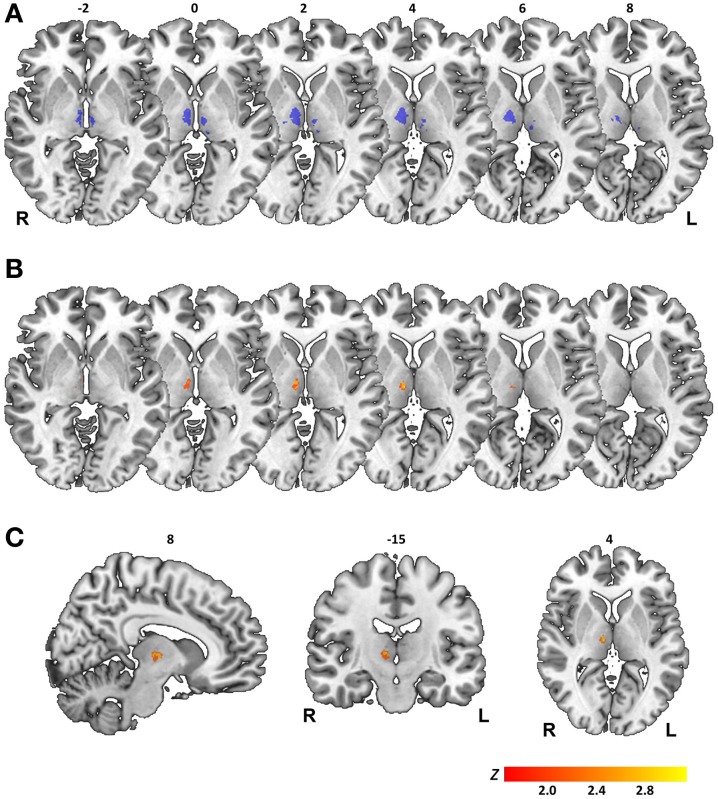
**Results of statistical voxelwise lesion-symptom mapping. (A)** Map of statistical power. Color-coded in blue are voxels where there is sufficient power to detect statistical effects at a threshold of *P* < 0.05, uncorrected. Map is shown on axial sections of a MNI brain template with numbers denoting *z*-coordinates in MNI space. L, Left; R, Right hemisphere. **(B)** Statistical map generated for threshold asymmetry in BLANK task is shown on corresponding axial sections. **(C)** Statistical map shown on sagittal, coronal, and axial sections with numbers denoting [*x*, *y*, *z*] coordinates of MNI space. Color scale in **(B,C)** indicates Z-scores (Liebermeister test), thresholded at *P* < 0.05 uncorrected.

For all three measures, overlay plots indicate a restricted portion of right central thalamus as region of maximum lesion overlap for impaired vs. unimpaired patients. For further illustration of the thalamic portion critical for task performance, we generated a statistical voxel-based lesion-symptom map, using non-parametric mapping (NPM) as implemented in MRIcron. This map was based on threshold asymmetry in the BLANK task (Figure 4E) as the behavioral measure of interest that yielded the highest degree of lesion overlap (Figure 6B). Figures 7B,C shows the resulting statistical map at a threshold of *P* < 0.05, uncorrected. Consistent with the three overlay images, this map identifies an area in right central thalamus as critical for the detection of intrasaccadic visual displacements. This area (cluster-size, 70 voxels) was centered at MNI-coordinates [8, −15, 4] and mostly encompasses lateral portions of MD nucleus, intralaminar nuclei, and medial parts of the ventrolateral nucleus. In principle, such an uncorrected map should be interpreted with caution, since it allows for the erroneous detection of false-positive findings. However, the individual lesion reconstruction with respect to an atlas of the human thalamus (see Figure 1B) allowed for a complementary analysis of affected nuclei in behaviorally impaired vs. unimpaired patients. This analysis confirmed a significant clustering of impaired performance with lesions of the right MD, CeM, and CL nuclei (Pearson's chi-square, all χ^2^ ≥ 4.7, *P* ≤ 0.03).

The map of statistical power shown in Figure 7A serves as reminder of a relevant limitation in our study population: lesion size in most patient cases was small and lesion topology biased toward medial and ventral portions of thalamus (see also lesion overlays, Figure 1A). This resulted in a limited coverage of both thalamic volumes where there was sufficient statistical power to detect a behavioral effect. Thus, while lesion-to-symptom mapping in our patient sample converges on a critical role of a restricted area of right central thalamus in predicting the visual consequences of saccadic eye movements, no firm conclusions can be drawn on a null contribution of left thalamus or more lateral, anterior, and posterior thalamic regions.

## Discussion

In the present study, we aimed to further elucidate the role of trans-thalamic CD pathways in the perceptual matching of visual input before and after an eye movement in human subjects. To this end, we used a visuomotor task requiring a perceptual judgment about the apparent direction of intrasaccadic stimulus displacements. We assessed performance in this task in a STEP and BLANK version, the latter allowing for high perceptual sensitivity in healthy subjects (Deubel et al., 1996). Accurate and precise perceptual decisions require matching of visual input before and after an eye movement and need to incorporate internal monitoring information of intervening eye movements, since oculomotor targeting errors render a sole reliance on visual errors insufficient.

Detection thresholds varied considerably in our comparatively large and heterogeneous group of healthy control subjects. However, individual threshold differences between conditions and saccade directions emerged as a more consistent measure across subjects (Bellebaum et al., 2005). Replicating previous findings, we noted a strong and highly significant improvement of detection performance in the BLANK compared to the STEP task for both saccade directions in healthy individuals (Deubel et al., 1996). Performance differences between these task variants might be driven by a differential weighting of internal monitoring with respect to visual reafferent information: Transient disappearance of the target in the BLANK condition might signal a possible violation of positional constancy to the visuomotor system (Deubel and Schneider, 1994; Deubel et al., 1996). Perceptual decisions might then more strongly rely on CD-driven internal predictions of visual reafference (Hamker et al., 2011). Consistent with this idea, non-stationary behavior of other stimulus features [such as stimulus motion (Gysen et al., 2002) or form changes (Demeyer et al., 2010)] has been shown to increase displacement detection across eye movements in a similar manner.

In recent case study (Ostendorf et al., 2010), we reported on a patient with a focal thalamic lesion who exhibited a lateralized deficit in the BLANK condition with an increased perceptual threshold relative to saccades in the other hemifield and relative to the STEP condition. Building on this case report, we contrasted performance in fourteen patients with focal thalamic lesions in different parts of the thalamus. We observed a similar impairment in perceptual performance in seven patients of our sample, with one patient failing to demonstrate a BLANK benefit, three patients exhibiting a large asymmetry between saccade directions and three patients demonstrating impairments for both of these behavioral measures (with the latter group including the single case reported previously).

Increased perceptual threshold in the single case mainly arose from a forward shift of the psychometric function (Ostendorf et al., 2010), i.e., from a systematic bias of the patient to report a backward jump of the target. This perceptual bias would be consistent with an internal underestimation of saccade amplitudes, e.g., a hypometric CD of corresponding oculomotor actions used for the perceptual judgment. Sign of this systematic error was consistent with oculomotor error patterns in rapid saccade sequences observed in this (Ostendorf et al., 2010) and other patients (Bellebaum et al., 2005) and in non-human primates after transient inactivation of CD transmitting relay neurons in central thalamus (Sommer and Wurtz, 2002). It also conformed to systematic changes of perceptual decisions induced by transcranial magnetic stimulation (TMS) over the FEF as the putative cortical target area of this trans-thalamic CD pathway (Ostendorf et al., 2012). In the current study, increased detection thresholds were caused by a forward shift of psychometric functions in the majority of individual cases. However, behavioral impairments manifested as backward shift of the psychometric function in three cases. Similar findings were reported by Gaymard et al. (1994) in two patients with thalamic stroke and suggest that disturbances of trans-thalamic CD transmission might also give rise to an internal overestimation of actual ocular state after eye movements. Oculomotor CD will pass through the thalamus as population code (Sanger, 2003). Partial damage to the pool of CD-transmitting neurons might then serve as possible explanation for idiosyncratic biases emerging on a perceptual level, depending on average saccade vectors represented by deficient vs. intact relay neurons.

Additional analyses of control subjects' data demonstrated that perceptual performance in the BLANK task was largely independent from oculomotor noise with no correlation between perceptual report and corresponding saccade targeting error for all but one subject and saccade direction. This further corroborates previous findings that healthy individuals can take CD information of oculomotor actions into account for their perceptual judgments (Collins et al., 2009). This would be expected if the visuomotor system is able to generate accurate and precise predictions of the visual error after saccade execution on a trial-by-trial basis, presumably by utilizing oculomotor CD (Guthrie et al., 1983). Mismatches between the internal prediction and actual reafference could then correctly be attributed to external stimulus displacements. By contrast, seven patients in the present study exhibited a systematic dependency of perceptual reports on oculomotor targeting errors. Apparently, these patients were not able to disambiguate self-induced targeting errors from external stimulus changes and consequently misattributed oculomotor errors to external stimulus changes.

Behavioral findings in our actual patient sample confirm that thalamic damage can indeed compromise the perceptual matching of space across eye movements and clearly suggests generalizability of the basic pattern of behavioral impairment previously observed in a single patient (Ostendorf et al., 2010). Beyond the issue of generalizability, single-case studies cannot speak to the anatomical specificity of brain-behavior relationships (Robertson et al., 1993). In this regard, findings of the actual study may aid to refine the mapping of behavioral impairments to a specific lesion topology. With a sizable number of patients being behaviorally unimpaired, acquisition of high-resolution imaging allowed for a voxel-based lesion-symptom mapping. The additional reconstruction of individual lesions with respect to an established atlas of the human thalamus (Morel, 2007) served as a second approach in lesion classification.

Lesion-symptom mapping in our patients converged on a restricted portion of the right central thalamus as critical for the matching of visual space across saccades. Following the nomenclature proposed by Morel (2007), this thalamic region comprised lateral portions of the MD nucleus (including the parvocellular and paralamellar division), central parts of intralaminar nuclei (mainly comprising the CL nucleus) and medial parts of the VL (i.e., ventral lateral posterior, VLp) and ventral posterior lateral nucleus (VPL). This thalamic region conforms well to the thalamic projection zone of pathways ascending from superior colliculus (SC) to the FEF identified in rhesus monkeys (Benevento and Fallon, 1975; Harting et al., 1980). It also complies with the anatomical reconstruction of intra-thalamic sites at which putative CD and eye position signals have been recorded in non-human primates (Schlag-Rey and Schlag, 1984; Schlag and Schlag-Rey, 1984; Sommer and Wurtz, 2004; Tanaka, 2007).

The region of central thalamus identified in our analysis is also largely consistent with previous patient studies that used the execution of rapid saccade sequences (Bellebaum et al., 2005) or intervening eye movements during the delay of a memory-guided saccade task (Gaymard et al., 1994) as a proxy to infer on internal updating mechanisms. In one of these studies, inspection of lesion topology suggests a more lateral location compared to the common lesion zone in our study, but involvement of central thalamic regions was presumed to underlie the behavioral deficit (Gaymard et al., 1994). In the other study (Bellebaum et al., 2005), lesions of behaviorally impaired patients were determined to affect the VL nucleus in three cases and the MD nucleus in another two cases. In this context, it seems important to note that positive evidence for a critical role of the right central thalamus in our study should not be taken as evidence against a possible role of left thalamus or more anterior, posterior and lateral thalamic portions for the perceptual matching of visual space across eye movements. Limited coverage in our patient sample precludes further inferences on a group level for these thalamic regions. Indeed, two patients that exhibited impairments in one behavioral measure in our study sustained focal and selective lesions of either the VL or ventral posterior (VP) nucleus, respectively.

With experimental evidence merged across human lesion studies, the lateralization of behavioral deficits with respect to thalamic lesion side remains largely equivocal so far. Inferring from the general organization of the visuomotor system, behavioral deficits would be expected for contraversive saccades after unilateral lesions of trans-thalamic CD pathways. In keeping with this notion, transient inactivation of functionally identified thalamic relay neurons led to a behavioral impairment for contraversive saccades in non-human primates (Sommer and Wurtz, 2002). Studies in human subjects with focal lesions in the thalamus yielded heterogeneous results with behavioral deficits manifesting contralateral (Gaymard et al., 1994; Bellebaum et al., 2005) and/or ipsilateral (Gaymard et al., 1994; Bellebaum et al., 2005; Ostendorf et al., 2010) to lesion side. Similarly, patients in our study exhibited behavioral deficits for both ipsiversive and contraversive saccade direction without any obvious relation of lateralization to lesion topology. In addition, bilateral behavioral deficits were not consistently associated with bilateral structural pathology.

Recent neurophysiological findings may at least partially account for these findings by demonstrating that the recipient area in frontal cortex receives oculomotor CD from both superior colliculi and hence all saccade directions (Crapse and Sommer, 2009). Moreover, CD information for both saccade directions is already present on the thalamic level (Tanaka, 2007) and might cross from the contralateral SC at tectal or thalamic levels (Crapse and Sommer, 2009). These findings in non-human primates might help to explain heterogeneous findings in human lesion studies, as partial lesions of CD pathways at the thalamic level might impair CD for some, but not all saccade directions. At present however, the spatial distribution of thalamic CD relay neurons is unclear and it is questionable whether a putative “saccadotopy” of CD within central thalamus might be sufficiently widespread and consistent across subjects to be picked up with MR imaging approaches.

Despite clear experimental evidence for a deficit in the internal monitoring of eye movements in seven of our patients, the subjective impression of visual stability was preserved in all our patients. This suggests compensatory mechanisms that operate efficiently outside the controlled setting of the laboratory. One likely factor contributing to the maintenance of visual stability may be the phenomenon of SSD itself (Bridgeman et al., 1975): general dampening of displacement detection across eye movements will attenuate the disturbing effect of saccade targeting errors on subjective perceptual continuity. Furthermore, relative positions between objects in a visual scene could be used as CD-independent source for a matching of visual space across eye movements (Gibson, 1966; Deubel, 2004). Oculomotor CD transmitted via the healthy thalamus and eye position information transmitted through spatially segregated thalamic relays (Gaymard et al., 2001; Sommer, 2003) represent candidate signals that might suffice to maintain perceptual coherence under these circumstances.

In conclusion, our results suggest that the integrity of central thalamus is critical for accurately predicting the visual consequences of eye movements. Successful disambiguation of self-induced vs. external changes in sensory input is central to adaptive behavior for various species and modalities (Crapse and Sommer, 2008). A global deficit in this elementary monitoring function has been presumed to contribute to debilitating symptoms in neuropsychiatric diseases, such as hallucinations and delusions of control (Feinberg, 1978; Fletcher and Frith, 2009). In this regard, findings in our study may serve as an exemplary instantiation of a circumscribed disturbance in such prediction mechanisms.

### Conflict of interest statement

The authors declare that the research was conducted in the absence of any commercial or financial relationships that could be construed as a potential conflict of interest.
